# Birth of Healthy Offspring following ICSI in *In Vitro-*Matured Common Marmoset (*Callithrix jacchus*) Oocytes

**DOI:** 10.1371/journal.pone.0095560

**Published:** 2014-04-21

**Authors:** Tsukasa Takahashi, Kisaburo Hanazawa, Takashi Inoue, Kenya Sato, Ayako Sedohara, Junko Okahara, Hiroshi Suemizu, Chie Yagihashi, Masafumi Yamamoto, Tomoo Eto, Yusuke Konno, Hideyuki Okano, Makoto Suematsu, Erika Sasaki

**Affiliations:** 1 Department of Applied Developmental Biology, Central Institute for Experimental Animals, Kawasaki-ku, Kawasaki, Kanagawa, Japan; 2 Department of Biochemistry, Keio University School of Medicine, Shinjuku-ku, Tokyo, Japan; 3 Department of Oncology, Juntendo University Nerima Hospital, Nerima-ku, Tokyo, Japan; 4 Marmoset Research Department, Central Institute for Experimental Animals, Kawasaki-ku, Kawasaki, Kanagawa, Japan; 5 Department of Biomedical Research, Central Institute for Experimental Animals, Kawasaki-ku, Kawasaki, Kanagawa, Japan; 6 Altair Corporation, Kohoku-ku, Yokohama-shi, Kanagawa, Japan; 7 Department of Physiology, Keio University School of Medicine, Shinjuku-ku, Tokyo, Japan; 8 Keio Advanced Research Center, Keio University, Shinjuku-ku, Tokyo, Japan; University of Hawaii at Manoa, John A. Burns School of Medicine, United States of America

## Abstract

Intracytoplasmic sperm injection (ICSI), an important method used to treat male subfertility, is applied in the transgenic technology of sperm-mediated gene transfer. However, no study has described successful generation of offspring using ICSI in the common marmoset, a small non-human primate used as a model for biomedical translational research. In this study, we investigated blastocyst development and the subsequent live offspring stages of marmoset oocytes matured *in vitro* and fertilized by ICSI. To investigate the optimal timing of performing ICSI, corrected immature oocytes were matured *in vitro* and ICSI was performed at various time points (1–2 h, 2–4 h, 4–6 h, 6–8 h, and 8–10 h after extrusion of the first polar body (PB)). Matured oocytes were then divided randomly into two groups: one was used for *in vitro* fertilization (IVF) and the other for ICSI. To investigate *in vivo* development of embryos followed by ICSI, 6-cell- to 8-cell-stage embryos and blastocysts were nonsurgically transferred into recipient marmosets. Although no significant differences were observed in the fertilization rate of blastocysts among ICSI timing after the first PB extrusion, the blastocyst rate at 1–2 h was lowest among groups at 2–4 h, 4–6 h, 6–8 h, and 8–10 h. Comparing ICSI to IVF, the fertilization rates obtained in ICSI were higher than in IVF (p>0.05). No significant difference was noted in the cleaved blastocyst rate between ICSI and IVF. Following the transfer of 37 ICSI blastocysts, 4 of 20 recipients became pregnant, while with the transfer of 21 6-cell- to 8-cell-stage ICSI embryos, 3 of 8 recipients became pregnant. Four healthy offspring were produced and grew normally. These are the first marmoset offspring produced by ICSI, making it an effective fertilization method for marmosets.

## Introduction

The common marmoset (*Callithrix jacchus*) is a small New World primate that has been used in biomedical research because of its physiological similarity to humans, its small body size, and its prolificacy. Marmosets show specific reproductive characteristics including a relatively short gestation period (about 144 days), reaching sexual maturity at 12–18 months, and female marmosets ovulate two or three oocytes in each ovarian cycle [1]. Utilization of this reproductive efficiency and the successful production of transgenic marmosets with germline transmission has been reported [2] and can be applied to genetically modified non-human primate models in life sciences [3].

*In vitro* production techniques of preimplantation embryos increase our understanding of the physiology of early embryonic development and improve animal production. In marmosets, *in vitro* fertilization (IVF) with fresh ejaculated sperm has achieved over 50% fertilization rates [4]–[6]. However, IVF requires high-quality and abundant sperm, which are difficult to obtain from infertile males, and it often gives rise to polyspermic embryos. To avoid these problems, intracytoplasmic sperm injection (ICSI) could be applied as an inseminating technique in marmosets.

The ICSI procedure has improved assisted reproduction technologies in rabbits [7], cattle [8], [9], mice [10], rhesus monkeys [11], and humans [12], [13], providing opportunities to investigate fundamental aspects of fertilization, such as the mechanisms of gamete interaction and sperm-induced oocyte activation. Furthermore, ICSI is a useful technique for efficient animal production of genetically modified animals or infertile male animals. This technology could be applied to transgenic animals via intracytoplasmic sperm injection-mediated transgenesis (ICSI-Tr) [14]. ICSI-Tr can be used to insert very large DNA fragments into the host genome. Currently, in non-human primates, the generation of transgenic animals has been reported using only lentiviral systems, which have limited insertional DNA sizes. ICSI-Tr can be applied to create various transgenic animal models, such as marmosets.

In marmosets, blastocyst stage embryos have been produced by ICSI and IVF using i*n vivo*-matured oocytes [5]. However, since the production of offspring from the ICSI embryos has not been reported, the developmental competence to neonate of ICSI embryos remains unknown. Furthermore, ICSI embryos from *in vitro-*matured oocytes have not yet been described.

Oocyte maturation depends on nuclear meiotic progression and is influenced by a quality and maturity of ooplasm [15], [16]. Previous studies observed frequent chromatin aberrations and decreasing developmental rates of the blastocyst stage when sperm insemination to oocytes was performed immediately after MII arrest [17], [18]. Therefore, essential cytoplasmic changes may occur during the MII arrest period, and successful embryo development depends on the proper timing of oocyte maturation, as well as oocyte fertilization.

This study aimed to determine the suitable timing of sperm injection into oocytes after *in vitro* maturation (IVM) and to investigate developmental competence of blastocysts of ICSI embryos using *in vitro*-matured oocytes in marmosets. Finally, we performed a quality evaluation of ICSI embryos using embryo transfer, reporting the first birth of a normal infant from ICSI embryos.

## Materials and Methods

### Animals

A total of 66 adult marmosets, purchased from a marmoset breeding company for experimental animals (CLEA Japan Inc., Tokyo, Japan), were used in this study. The body weights and ages of the marmosets ranged from 284–534 g and 2–8 years, respectively. The animals were not sacrificed for the current experiments. All animal experiments were approved by the Institutional Animal Care and Use Committee of the Central Institution for Experimental Animals (CIEA) (CIEA approval no: 11028A) and were performed in accordance with the CIEA guidelines that agree with the Guidelines for Proper Conduct of Animal Experiments by the Science Council of Japan (2006). Animal care was conducted in accordance with the recommendations of the Guide for the Care and Use of Laboratory Animals (Institute of Laboratory Animal Resources, 1996). The marmosets were housed in pairs in stainless steel living cages (39×55×70 cm) with wire mesh floors maintained at 25–26°C with 45–55% humidity and illumination for 12 h per day. Wood perches for locomotion and gouging and a platform for a bed were placed in each cage for environmental enrichment. Marmosets were kept healthy and well-nourished with a balanced diet (CMS-1M; CLEA Japan Inc.), including mixed L (+)-ascorbic acid (Nacalai Tesque, Tokyo, Japan), vitamins A, D_3_, and E (Duphasol AE_3_D; Kyoritsu Seiyaku Co., Ltd., Tokyo, Japan), and honey (Nihonhatimitsu Co., Ltd., Gifu, Japan). In addition, chicken liver boiled in water (DBF Pet Co., Ltd., Niigata, Japan) was given as a supporting meal once a week. The animals were supplied with tap water *ad libitum* from feed valves.

### Anesthesia and Postoperative Care

Animals were pre-anesthetized with an intramuscular injection of 0.04 mg/kg of medetomidine (Domitor; Nippon Zenyaku Kogyo, Koriyama, Japan), 0.40 mg/kg of midazolam (Dormicam 10 mg; Astellas Pharma, Tokyo, Japan), and 0.40 mg/kg of butorphanol (Vetorphale; Meiji Seika Pharma, Tokyo, Japan). They were also administered 15 mg/kg ampicillin (Viccillin; Meiji Seika Pharma, Co., Ltd.) and hydrated subcutaneously with 2 mL/head of fluid (KN No.1 injection; Otsuka Pharmaceutical, Tokyo, Japan). Thereafter, animals were anesthetized by inhalation with 1.0–3.0% isoflurane (Forane; Abbott Japan, Tokyo, Japan) via a ventilation mask. Anesthetization management was performed by spontaneous respiration during the operation, monitoring the heart rate and the arterial oxygen saturation. After oocyte collection or embryo transfer, 0.20 mg/kg atipamezole (Antisedan; Nippon Zenyaku Kogyo) was administered intramuscularly into the animals. For postoperative analgesia and infection control, 1.2 mg/kg ketprofen and 15 mg/kg ampicillin were administered once daily for 3 consecutive days following the operations.

### Ovarian Stimulation and Collection Oocytes

Thirty-three donor female adult marmosets were used in the present study. For oocyte collection from ovaries, donor female marmoset ovarian stimulation was performed using the follicular stimulation protocol, as described previously [2]. Briefly, ovarian cycles were monitored based on plasma progesterone levels using an enzyme immunoassay (EIA) kit (TOSO Progesterone Kit; TOSO, Tokyo, Japan). Luteolysis was induced with 0.8 µg of cloprostenol, an analog of prostaglandin F_2α_ (PGF_2α_; Estrumate; Schering-Plough Animal Health, Union, NJ), which was administered by intramuscular injection. After injection, onset of the follicular phase was confirmed based on blood progesterone levels the day after PGF_2α_ injection. Marmoset follicles were stimulated by intramuscular injection of recombinant human follicle-stimulating hormone (rhFSH; 50 international units (IU); FOLYRMON – P injection; Fuji pharma Co., Ltd, Tokyo, Japan) for 9 days at 10∶00, and human chorionic gonadotropin (hCG; 75IU; Gonatropin; ASKA Pharmaceutical. Co. Ltd., Tokyo, Japan) was administered on the day of the ninth FSH administration at 17∶30. Thirty hours after hCG injection, animals were anaesthetized as described above and follicular aspiration was performed. Oocytes were collected in porcine oocyte medium (POM; Research Institute for the Functional Peptides, Yamagata, Japan) [19].

### *In Vitro* Maturation (IVM)

Collected oocytes were washed three times with mPOM (POM supplemented with 5% heat-inactivated calf serum (FBS; Gibco, Carlsbad, CA) and 100 IU/mL FSH (Folyrmon-P; FujiPharma, Tokyo, Japan). *In vitro* oocyte maturation was performed by incubation in an 80 µL mPOM drop covered with mineral oil (Nacalai Tesque, Tokyo, Japan) under a gas phase of 5% CO_2_, 5% O_2_, and 90% N_2_ at 37.5°C. To measure the suitable ICSI timing after the first PB extrusion, cumulus cells were degraded with 1 mg/mL hyaluronidase (Sigma) for 1 min after 19 h of IVM, oocytes were changed to independent cultures and the first PB extrusion observed was used in microscopy every 30 min. The time of the first PB extrusion was recorded for each oocyte and divided randomly into five groups 1–2 h, 2–4 h, 4–6 h, 6–8 h and 8–10 h followed by ICSI.

When comparing ICSI with IVF, cumulus cells were partially removed by gentle pipetting after 26 h of IVM and the first PB extrusion was evaluated. The oocytes from each animal were divided into two groups, IVF and ICSI. For ICSI, the cumulus cells were completely degraded with hyaluronidase. Twenty-seven hours after the IVM, ICSI and IVF were performed.

### Sperm Collection and Preparation

Eight male adult marmosets were used in the present study. Of the eight male animals, three were selected based on their physical conditions for each experiment. Marmoset semen was collected as described previously [20]. The animal was restrained in an upright position, and penile vibratory stimulation was performed using a FertiCare personal vibrator (Fertility Healthcare and Supplies, Inc., Silverado, CA, USA) in three sexually mature donor animals. Pre-equilibrated 700 µL TYH medium (Mitsubishi Chemical Medience Corp., Tokyo, Japan) was immediately added to the ejaculate and the suspension was incubated at 37.5°C for approximately 30 min to disperse sperm from the coagulum. The semen was washed twice with 700 µL of TYH medium by aspiration of the supernatant after centrifugation for 5 min at 400×*g*, after which 200 µL of fresh TYH medium was added. The resuspended sample was then supplied to the bottom of a conical tube containing 500 µL fresh TYH. A total of 700 µL TYH with sperm suspension was incubated for 30 min at 37.5°C under a gas phase of 5% CO_2_, 5% O_2_, and 90% N_2_ to allow the sperm to swim upwards. After incubation, 400 µL of supernatant was collected and the quality of sperm samples was measured using the Sperm Motility Analysis System (SMAS; Ditect, Tokyo, Japan) based on viability, motility, and concentration. Samples of the highest quality were chosen for IVF and ICSI, and the sperm suspension was divided into two samples. For the IVF study, the sperm suspension was adjusted to a final sperm concentration of 3.6×10^6^ sperms^/^mL and then into 30 µL drops. For the ICSI study, the sperm suspension was washed with TYH and centrifuged for 5 min at 400×*g*. After washing, the sperm pellet was resuspended in M2 medium (Sigma, St. Louis, MO) and adjusted to a final sperm concentration of 1×10^4^ sperms/mL.

### Intracytoplasmic Sperm Injection (ICSI)

An oil-covered micromanipulation chamber containing a 20 µL drop of M2 for oocytes and a 10 µL drop of M2 medium containing 10% polyvinyl pyrrolidone (PVP; Sigma) for spermatozoa was prepared. Approximately 1 µL of sperm suspension was transferred into M2 medium containing 10% (w/v) PVP drops and mixed thoroughly. One to three oocytes were placed in the M2 medium drop. Using an inverted microscope with micromanipulators (Narishige, Tokyo Japan), only motile sperm with normal morphology were selected and immobilized by pressing the tail with the injection needle tip against the dish bottom prior to injection. A single sperm was aspirated from the sperm drop and moved to a droplet containing oocytes. An oocyte was captured by the holding pipette and immobilized with its PB at either the 6 or 12 o’clock position, and the zona pellucida was drilled using piezo pulses. The pipette was inserted deeply into the oocyte and a single piezo pulse was applied. A spermatozoon was then injected into the cytoplasm. After ICSI, oocytes were placed in ISM1 medium drops (Medicult; Nosan Corp., Kanagawa, Japan) and washed three times. The day of performing ICSI was designated as day 0.

### *In Vitro* Fertilization (IVF)

After sperm preparation, the matured oocytes were washed three times with 50 µL of TYH medium drops. The oocytes were transferred into 30 µL drops of TYH medium containing 3.6×10^6^ sperms/mL and incubated for 18 h under a gas phase of 5% CO_2_, 5% O_2_, and 90% N_2_ at 37.5°C. After 18 h, embryos were washed three times with 70 µL ISM1 drops. The day of performing IVF was designated as day 0.

### *In Vitro* Culture (IVC)

Human embryo culture medium ISM 1 and 2 (ORIGIO, Måløv Denmark) were used in this study to culture marmoset embryos. Pronuclear formations of embryos followed by IVF or ICSI were confirmed under microscopic observation. Embryos were transferred in 70 µL fresh drops of ISM1 and cultured under mineral oil cover at 37.5°C in 5% CO_2_, 5% O_2_, and 90% N_2_ for 3 days. On day 3, embryos were transferred to 70 µL of ISM2 drops covered with mineral oil and were placed in the incubator at 37.5°C in a humidified atmosphere of 5% CO_2_, 5% O_2_, and 90% N_2_ until day 12. The media exchange and checking of embryo development were performed every 2 days. The embryos that developed to blastocysts were examined at days 9, 10, and 11.

### Non-surgical Embryo Transfer

Twenty-eight recipient female adult marmosets were used in the present study. The ovarian cycles of donor and recipient animals were synchronized using PGF_2α_, and ovarian cycles were monitored based on plasma progesterone levels. The embryos produced by ICSI were transferred to surrogate mothers using nonsurgical embryo-transfer techniques, as described previously with modified instruments [21]. The three different diameter-sized, 7.5-cm-long glass tubes were newly developed for the noninvasive embryo transfer technique. The thinnest glass tube size was tapered to 4.0 mm with a 4.2 mm diameter, the middle size glass tube was tapered to 5.0 mm with a 6.0 mm diameter, and the widest size glass tube was tapered to 5.3 mm with a 7.0 mm diameter at one end. The Fluon ETFE 20-gauge (G) was comprised of a cannula 108 mm in length (a blunt/tapered cannula; 0.8-mm inner diameter and 1.10-mm outer diameter; Oviraptor; Altair Corp., Yokohama, Japan). The 23-G had 120-mm-long blunt-end stainless steel stylets and a polyethylene 160-mm-long cannula (inner diameter 0.28 mm and outer diameter 0.61 mm; Oviraptor; Altair Corp.). These devices were developed to reduce invasiveness to the animals and improve the insertion of the cannula into the uterus. All instruments were sterilized before use with a hydrogen peroxide gas plasma sterilizer (Sterrad 50; Advanced Sterilization Products, Irvine, CA), and the surgeon and surgical assistants wore sterile surgical gloves.

Vaginal dilation of the anesthetized recipients was performed gradually by serial introduction and removal of three sizes of 75-mm-long glass tubes (from thinnest to widest). The widest glass tube was placed in the vagina to manipulate the endoscope and cannula to prevent vaginal injury. An endoscope (1.6 mm in diameter; TESALA AE-C1; AVS Co., Ltd., Tokyo, Japan) was inserted into the glass tube to observe the ostium uteri externum. A blunt/tapered Fluon ETFE 20-G outer cannula combined with a 23-G, 120-mm-long blunt-end stainless steel stylet were inserted into the cervix via the glass tube. After inserting the Fluon ETFE 20-G outer cannula/23-G blunt-end stainless steel stylet into the uterus, the blunt inner stainless steel stylet was removed. At this time, the polyethylene cannula was inserted into the Fluon ETFE 20-G outer cannula as the dummy inner cannula. The uterus was observed using linear ultrasound probe (Prosound α7: Hitachi Aloka Medical, Ltd., Tokyo, Japan) by longitudinally placing it onto the abdomen to confirm insertion of the dummy inner cannula via the outer cannula into the uterus. Approximately 2 µL of medium containing one to three embryos was loaded into a new polyethylene cannula that attached to a 50-µL glass syringe (Hamilton Co., Reno, NV). After removing the dummy inner cannula, the inner catheter containing the embryos was inserted into the outer cannula. When the inner catheter was approximately 3 mm from the distal end of the uterus, the outer cannula was pulled back until the cannula tip reached the proximal end of the uterus from the cervix. The embryos were then delivered into the uterine lumen by gentle pressure on the glass syringe. The cannula and catheter were withdrawn after embryo transfer and washed with medium to confirm that no embryos remained in the cannula and catheter.

The recipients were tested for pregnancy based on plasma progesterone measurements once a week until the time at which pregnancy could be monitored using ultrasonography in the uterus.

The blastocyst stage embryos underwent embryo transfer 12 days after ICSI and the 6-cell- to 8-cell-stage embryos were transferred into a surrogate mother uterus at 5 days after ICSI.

### Genotyping of Neonates Using Microsatellite Markers

Parental testing based on microsatellite polymorphisms for the delivered offspring was performed using 10 microsatellite markers (Table 1). Genomic DNA was extracted from hair root for live animals or frozen skin for dead animals using the DNA Micro Kit (Qiagen KK, Tokyo, Japan). To detect microsatellite polymorphisms, polymerase chain reaction (PCR) amplification was performed using the extracted genomic DNA as template. The PCR product was loaded directly on an ABI 3130×l Genetic Analyzer (Life Technologies, NY, USA) along with the GS500 LIZ dye Size Standard (Life Technologies, NY, USA). The electrophoresis data was processed using GeneMapper 4.0 software (Life Technologies), and alleles were assigned according to the PCR product size.

**Table 1:** List of primers and their sequences in multiplex PCR.

Name	Sequence of the labeled primer (5′–3′)	Dye	Name	Sequence of the non-labeled primer (5′–3′)
2463P-TH	GCACAGGCAGATTCAAGACAACTC	FAM	2463P-TH-NL	CCAAGACCTCAGGGAGGTAGTAGG
CJ060	TGCTCTAGAGGTTCCACTCTG	PET	CJ060-NL	GGCATGTTACCTAACCTCTCTG
CJ077	ATTCCATTCTGGGCAGCAAG	PET	CJ077-NL	CCTCCCATACTACAGATGAGGA
CJ081	TTCCCCTCTCTTTCAGACACA	VIC	CJ081-NL	CACCTCCTCTTCAAGTAAACACC
CJ103	CCCTTTCCTGCTAATTCACAGAAG	NED	CJ103-NL	CTGGGTAACAAGAGTGAAACTCC
CJ187	TGGAAGAACTTTCTGCCAAACC	FAM	CJ187-NL	GCTTGTTCAGGCAGACTGAC
CJ003	AGATGTGGCAGTTGTCTTGG	NED	CJ003-NL	TCTCTGCCATAGTGACCTCT
CJ083	TTGTACCCTTTTGCTTGCAG	VIC	CJ083-NL	TTCCTTCTTTTGGGGAGTGT
CJ091	CCTGCACCCGTAAATAGGTTC	FAM	CJ091-NL	CATCCTGGGCAACAAGAGTG
CJ146	CTTAATTCTGCCACAGTAGCAC	PET	CJ146-NL	GAGAGTCCCTAAATGCAAGGA

### Statistical Analysis

To evaluate differences between experimental groups, a χ^2^ - test was performed. Differences at p*<*0.05 were considered significant.

## Results

### Fertilization Competence after PB Extrusion

**Figure 1:**
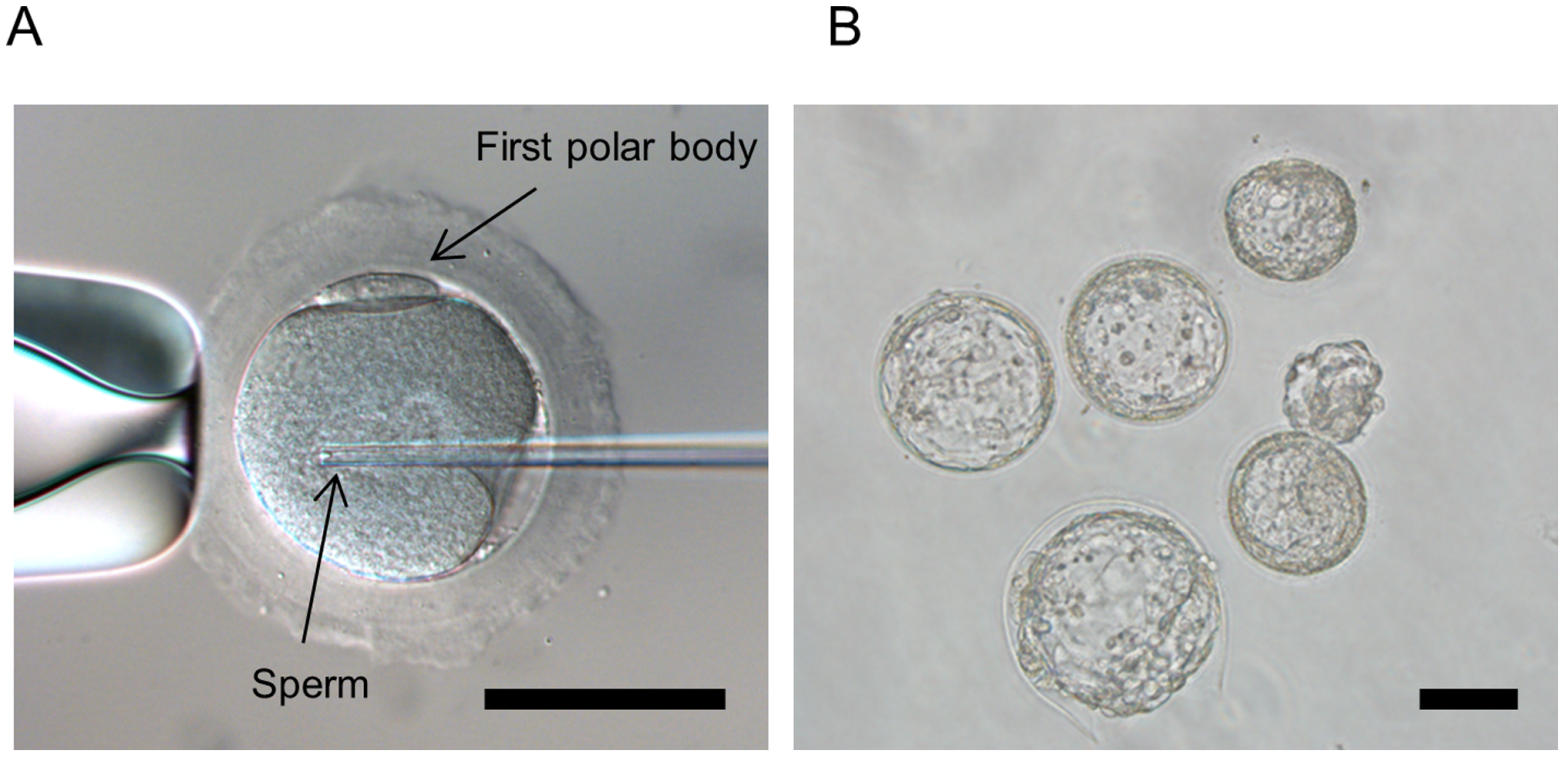
ICSI of marmoset oocytes after *in vitro* maturation. (A) The zona pellucida was drilled using piezo pulses, and the pipette was inserted deep into the oocyte and a single piezo pulse was applied. Sperm were inserted into the oocyte (bar = 100 µm). (B) Marmoset blastocysts produced by ICSI. (bar = 100 µm).

PB extrusions were observed approximately 20–24 h after IVM. To determine the optimal timing of performing ICSI, 104 *in vitro*-matured oocytes derived from 12 female marmosets were divided into five groups and subjected to ICSI at various time points: 1–2 h, 2–4 h, 4–6 h, 6–8 h, and 8–10 h after extrusion of the first PB (Figure 1A, Table 2). Although no significant differences were observed in the fertilization and blastocyst rate, the blastocyst rate tended to be low among the groups when ICSI was performed at 1–2 h after PB extraction (5.0%, 31.6%, 16.0%, 26.7%, and 20.0%, respectively).

**Table 2:** Fertilization and developmental rates after ICSI of *in vitro*-matured marmoset oocytes.

ICSI time post-firstPB extrusion	No. of oocytes	No. (%)* ^1^ offertilized oocytes	No. (%) * ^2^ of embryos developed to		
			2-cell	8-cell	Blastocyst
1–2 h	23	20 (87)	18 (90)	14 (70)	1 (5)
2–4 h	20	19 (95)	18 (94.7)	16 (84.2)	6 (31.6)
4–6 h	25	25 (100)	25 (100)	20 (80)	4 (16.0)
6–8 h	16	15 (93.8)	15 (93.8)	13 (86.7)	4 (26.7)
8–10 h	20	20 (100)	20 (100)	16 (80)	4 (20)

*^1^Numbers in parentheses were calculated from total oocytes.

*^2^Numbers in parentheses were calculated from fertilized oocytes.

### Developmental Competence of Oocytes Fertilized by ICSI or IVF

To compare embryo developmental competencies, ICSI and IVF were performed (Table 3). Since relatively high embryo development was shown beyond 2 h after PB extrusion, ICSI and IVF were performed after 27 h following IVM that fit to 3–7 h after PB extrusions. In total, 178* in vitro*-matured oocytes derived from 21 female marmosets were divided into two groups for ICSI or IVF. The fertilization and developmental rates during the blastocyst stage of ICSI and IVF embryos are shown in Figure 1B and Table 3. The fertilization rate of the ICSI embryos (93.2%) was significantly higher than that of the IVF embryos (82.2%, p<0.05). No significant differences in developmental rate were observed between ICSI and IVF embryos (35.4% and 39.2%, respectively) in the blastocyst stage.

**Table 3:** Effect of fertilization method on *in vitro* development of marmoset oocytes.

	No. of maturedoocytes	No. (%)* ^1^ ofFertilized oocytes	No. (%) * ^2^ of embryos developed to				
			2-cell	8-cell	16-cell	Morula	Blastocyst
ICSI	88	82(93.2)^a^	80(97.6)	63(76.8)	43(52.4)	32(39.0)	29(35.4)
IVF	90	74(82.2)^b^	69(93.2)	62(83.8)	48(64.9)	36(48.6)	29(39.2)

Values within the same column with different letters (a, b) differ significantly (p<0.05), χ^2^-test.

*^1^Numbers in parentheses were calculated from total oocytes.

*^2^Numbers in parentheses were calculated from fertilized oocytes.

The length to reach the blastocyst stage of embryos using ICSI and IVF are shown in Table 4.

**Table 4:** The length of embryonic development until the blastocyst stage of the embryos derived by ICSI and IVF.

	No. of total blastocyst	No. (%) * of embryos developed to blastocyst		
		Day 9	Day 10	Day 11
ICSI	29	6 (20.7)	9 (31.0)	14 (48.3)
IVF	29	9 (31.0)	10 (34.5)	10 (34.5)

*Numbers in parentheses were calculated from total blastocysts.

Day 0: Day of fertilization.

Although the developmental speed to blastocyst was not significantly different, ICSI embryos tended to develop later than IVF embryos.

### Developmental Abilities of the Embryos to Neonates

**Figure 2:**
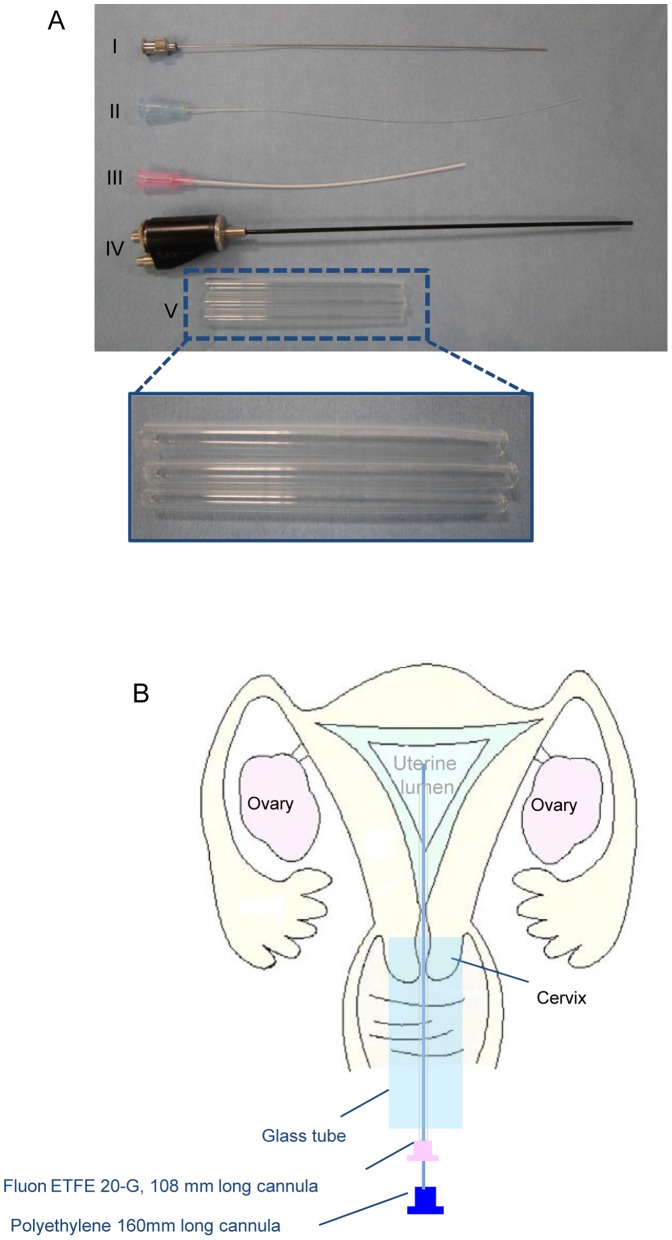
Devices for nonsurgical embryo transfer. Newly developed devices for nonsurgical embryo transfer. (A) I: 23-G, 120-mm-long blunt-end stainless steel stylet II: polyethylene 160-mm-long cannula (inner diameter 0.28 mm, outer diameter 0.61 mm) for embryo transfer, III: Fluon ETFE 20-G, 108-mm-long cannula (A blunt/tapered cannula; inner diameter 0.8 mm, outer diameter 1.10 mm), IV: endoscope for small animals, V: tapered to 4.2 mm, 6.0 mm in diameter and tapered to 5.0 mm, 7.0 mm in diameter and tapered to 5.3 mm at one end of the glass tubes for vaginal dilation and manipulation of the cannulae. (B) Scheme for nonsurgical embryo transfer.

**Figure 3:**
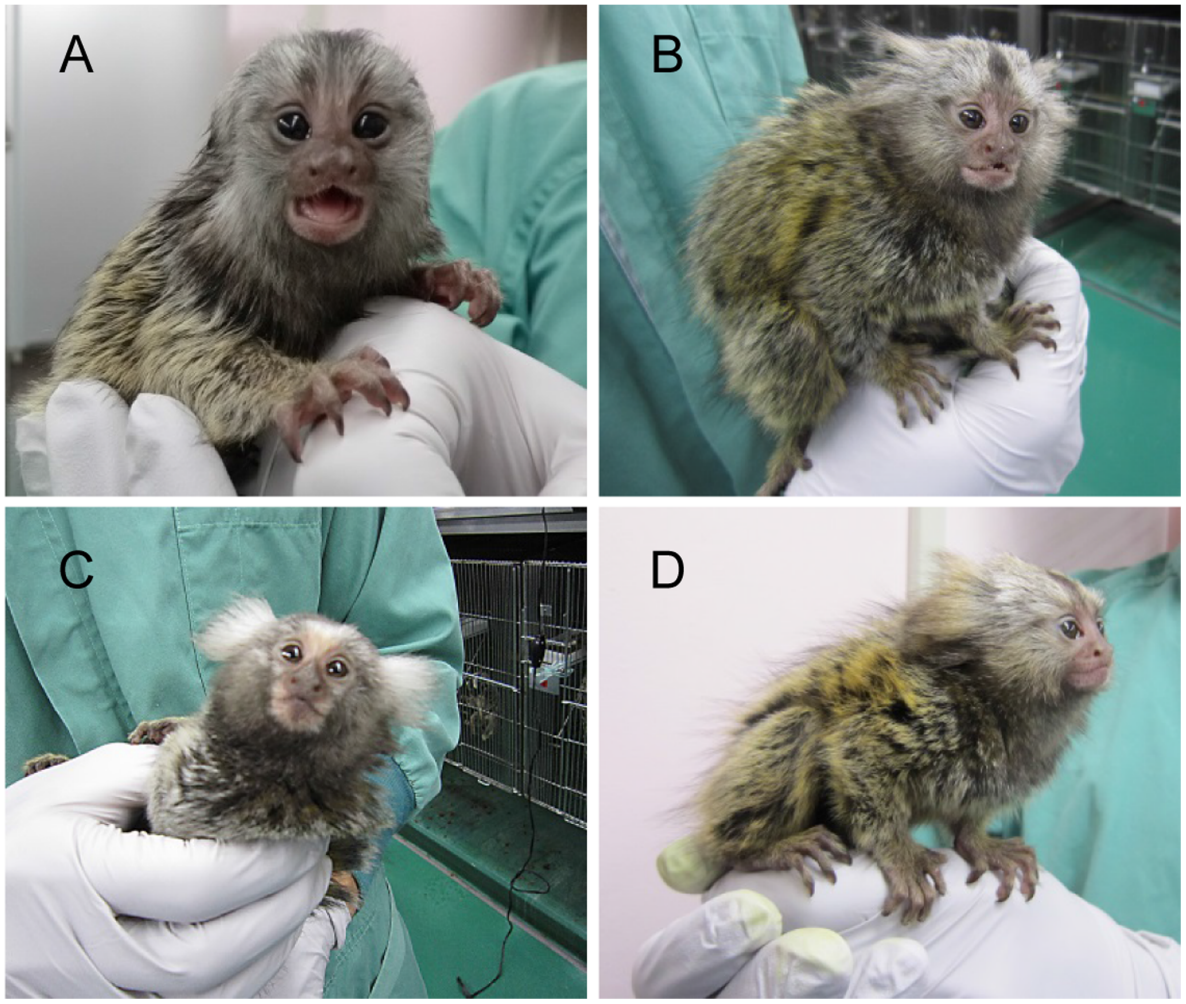
First offspring produced by ICSI in marmosets. Four healthy neonates were generated using ICSI. A female (A) 635 produced by blastocyst transfer and a male neonate (B) 732, male (C) 737 and female neonate (D) 640 from 6-cell- to 8-cell stage embryo transfer.

To assess the *in vivo* developmental potential of ICSI-derived blastocysts (Figure 1B), 37 blastocysts after 12 days of culture were transferred into 20 recipient surrogate mothers by nonsurgical embryo transfer (Figure 2). Four of 20 recipients receiving blastocysts produced by ICSI were pregnant. Although one normal and healthy offspring (Figure 3, Table 5, Table 6) was delivered, other recipients interrupted pregnancy by spontaneous abortion at days 83, 104, and 105. Therefore, to investigate whether the *in vivo* developmental potential of relatively early ICSI embryos was higher, 21 embryos from the 6-cell- to 8-cell stage after 5 days of culture were transferred into eight recipients (Table 5). We found that three of eight recipients were pregnant and six offspring were delivered. Although three offspring were dead a few days after delivery by caesarean section, the other three offspring grew to be healthy. The birth rate of the 6-cell- to 8-cell-stage embryo transfer was 28.6%, which was significantly higher than that of the blastocyst stage embryo transfer (2.7%, p<0.05).

**Table 5:** Pregnancy and birth rates following nonsurgical embryo transfer to recipients of common marmoset embryos produced by ICSI.

Embryo age: stage attransfer	No. of Embryostransferred	No. of recipients	No. (%)* ^1^ of pregnant recipients			No. (%)* ^2^ ofoffspring born	No. (%)* ^2^ of deadoffspring after birth
			Total	Delivered	Aborted		
6-cell–8-cell (day 5)	21	8	3 (37.5)	3 (37.5)	0 (0)	6 (28.6)^a^	3 (14.3)
Blastocyst (day 12)	37	20	4 (20)	1 (5)	3 (15)	1 (2.7)^b^	0 (0)

Values within the same column with different letters (a, b) differ significantly (p<0.05), χ^2^-test.

*^1^Numbers in parentheses were calculated from total recipients.

*^2^Numbers in parentheses were calculated from total embryos.

**Table 6:** Details of offspring produced by ICSI.

Transferred embryo(stage/numer)	The neonates ID	Body weight (g)	Status after birth
Blastocyst/2	635	36	alive
8-cell/3	731	27.7	dead on day 5
	732	25.6	alive
	733	24.6	dead on day 16
	734	ND	dead on day 1
8-cell/2	737	32	alive
8-cell/2, 6-cell/1	640	32	alive

### Parentage Evaluation Tests Using Microsatellite Markers

The results of genotyping of neonates using microsatellite markers clearly demonstrated that all offspring were derived from donor embryos (Table 7). For offspring 635, the genotype with CJ060-PET, CJ077-VIC, CJ081-VIC, CJ103-NED, CJ003-NED, and CJ083-VIC microsatellite markers demonstrated that this neonatal animal was derived from donors 3464 and 691. The genotype with CJ060-PET, CJ081-VIC, and CJ103-NEDmicrosatellite markers indicated that offspring 731, 732, 733 and 734 was not derived from the recipients, but rather from donors 3525 and 666. Similarly, the paternity testing indicated that offspring 737 and 640 were from the donor animals.

**Table 7:** Analysis of microsatellite genotypes of donors, recipients, and offspring in embryo transfer of marmoset embryo produced by ICSI.

			Marker* ^1,^ * ^2^									
Pedigree	The neonates ID	Sex	2463-TH-FAM	CJ060-PET	CJ077-VIC	CJ081-VIC	CJ103-NED	CJ187-FAM	CJ003-NED	CJ083-VIC	CJ091-FAM	CJ146-PET
#1	Recipient 2370	Female	109/109	135/146	205/205	170/184	119/121	201/201	90/94	119/125	139/145	134/134
	Recipient 3298	Male	109/109	146/151	205/213	184/186	119/121	201/207	94/94	119/121	139/139	132/134
	Donor 3464	Female	109/109	**135**/**135**	**205**/**205**	160/**168**	111/**121**	201/201	94/**96**	**121**/**121**	139/139	132/134
	Donor 691	Male	109/109	**135**/**135**	205/**209**	168/**186**	**107**/117	201/207	**94**/**94**	115/**121**	139/139	132/134
	Offspring 635	Female	109/109	**135**/**135**	**205**/**209**	**168** / **186**	**107**/**121**	201/207	**94**/**96**	**121**/**121**	139/139	134/134
#2	Recipient 2343	Female	109/109	135/135	205/211	160/186	117/117	201/201	94/98	121/121	139/139	132/132
	Recipient 659	Male	109/109	149/151	213/213	170/170	111/121	201/209	94/96	121/125	139/147	134/134
	Donor 3525	Female	109/109	135/**137**	205/213	160/**186**	119/**125**	201/209	94/94	121/121	139/139	132/134
	Donor 666	Male	109/109	**135**/**135**	205/213	**168**/**186**	119/**121**	201/201	94/98	121/121	139/139	134/134
	Offspring 731	Male	109/109	**135**/**137**	213/213	**186**/**186**	**121**/**125**	201/201	94/98	121/121	139/139	132/134
	Offspring 732	Male	109/109	**135**/**137**	205/213	**168**/**186**	**121**/**125**	201/209	94/98	121/121	139/139	134/134
	Offspring 733	Male	109/109	**135**/**137**	213/213	**186**/**186**	**121**/**125**	201/201	94/98	121/121	139/139	132/134
	Offspring 734	Male	109/109	**135**/**137**	213/213	**186**/**186**	**121**/**125**	201/201	94/98	121/121	139/139	132/134
#3	Recipient 2706	Female	109/109	135/146	205/205	170/184	117/125	201/201	90/94	119/125	139/145	134/134
	Recipient 3346	Male	109/109	135/139	205/205	184/186	117/117	201/201	94/98	119/121	139/139	134/134
	Donor 2703	Female	109/109	135/146	205/205	**168**/186	107/**115**	201/207	94/98	**121**/**121**	139/139	132/134
	Donor 666	Male	109/109	135/135	205/213	**168**/186	**117**/119	201/201	94/98	**121**/**121**	139/139	134/134
	Offspring 737	Male	109/109	135/135	205/205	**168**/**168**	**115**/**117**	201/201	94/94	**121**/**121**	139/139	134/134
#4	Recipient 3196	Female	109/109	135/137	205/205	160/170	107/121	201/201	90/94	121/123	139/139	132/134
	Recipient 3343	Female	109/109	135/135	203/205	168/168	111/121	201/201	94/98	121/123	139/139	132/134
	Donor 2703	Female	109/109	135/**146**	205/205	**168**/186	**107**/115	201/207	94/98	121/121	139/139	132/134
	Donor 666	Male	109/109	**135**/**135**	205/213	**168**/186	**117**/119	201/201	94/98	121/121	139/139	134/134
	Offspring 640	Female	109/109	**135**/**146**	205/205	**168**/**168**	**107**/**117**	201/201	94/94	121/121	139/139	134134

*^1^Size in bp.

*^2^Underlined numbers indicate unique alleles inherited from the donor, and bold numbers indicate the genotypes used to determine whether the offspring were derived from donor animals.

## Discussion

The present study is the first to report the birth of common marmoset offspring using ICSI with oocytes matured *in vitro*. Based on the results of genotyping tests using microsatellite markers, all neonates were derived from the ICSI embryos.

Since ICSI can control the timing of fertilization, the optimal fertilization timing through oocyte maturation was determined. Oocyte nuclear maturation implies re-initiation and completion of the first meiotic division from the GV stage to MII stage. Besides these nuclear aspects of oocyte maturation, cytoplasmic aspects are also important for fertilization and development of the oocyte [22]. These two processes are completely independent events [22]–[25]. The study of human, mouse, and bovine oocytes indicated that cytoplasmic maturation may occur during MII arrest [17], [18]. In humans, IVM oocytes require at least 1 h incubation for cytoplasmic maturation after the first PB extrusion to fertilize by ICSI [26], [27]. In this study, the fertilization and embryonic developmental rates of the marmoset ICSI embryos after IVM oocytes at various time intervals following extrusion of the first PB showed no significant differences. Although significant differences were not observed, the blastocyst rate in 1–2 h groups was lowest among the groups, suggesting that the optimal timing of fertilization was more than 2 h after PB extrusion in marmosets. In this study, both ICSI and IVF were performed after 27 h following IVM, which were adjusted to 3–7 h after PB extrusion, and both embryos showed comparable developmental rates. Furthermore, the observation of marmoset oocyte PB extrusions approximately 20–24 h after IVM (data not shown) was consistent with previous reports [4]. The ICSI procedure bypasses the normal fertilization process through IVF of zona penetration and fusion of sperm and oocyte membranes. Thus, the initial steps involved in oocyte activation may also be bypassed. In several species such as mice, humans, and rabbits, ICSI can activate oocytes for further embryonic development, comparable to IVF [10], [28], [29]. In bovine and porcine embryos, the developmental competencies of embryos followed by ICSI were low. To improve the development of embryos, ICSI has been combined with artificial stimuli, such as exposure to ethanol, ionomycin, 6- dimethylaminopurine (DMAP), and electric stimulation. [30], [31]. Our results demonstrated that highly efficient marmoset fertilization and blastocyst development occurred using ICSI with no stimulation, indicating that sperm injection alone is sufficient to activate oocytes, similar to that of humans, mice and rabbits.

Although the amount of total blastocysts between IVF and ICSI was not significantly different, the time for embryos to reach the blastocyst stage tended to be longer when using ICSI than IVF. In our previous study, IVF embryos showed a 12% birth rate after embryo transplantation [19]. In the present study, the birth rate following the transplantation of ICSI embryos was 28.6%. Although experimental conditions in the previous study differed from this study in two aspects, (POM medium without FBS and embryo transfer method), the birth rate of ICSI and IVF embryos was similar.

Our results indicate that the birth rate from cleavage-stage embryo transfers was significantly higher than that of blastocyst transfers. Three of six offspring obtained from 6-cell- to 8-cell-stage transfer were dead after birth. These three offspring were quadruplets and derived by caesarean section. However, three of four offspring were dead on the first day, day 5, and day 16 after birth, respectively. The average weight of these offspring was 25.6 g lower than the average weight of normal neonates (33.3 g). Thus, the postnatal deaths of these neonates did not result from low embryo quality, but from premature birth of the quadruplets. These quadruplets were obtained from a recipient female that received three embryos. Parental testing demonstrated that all offspring were from donor embryos. Interestingly, offspring #731, #733 and #734 showed the same genotyping and may have been identical triplets, indicating that one ICSI embryo split in the recipient uterus. One reason for the low birth rate of blastocyst embryo transfer may have been the long-term embryo culture *in vitro*. In this study, human embryo culture mediums ISM 1 and 2 were used to culture marmoset embryos. We examined various human IVC mediums using *in vivo* fertilized marmoset embryos collected from female marmosets’ uteri and chose ISM2, which showed the highest developmental ability to the blastocyst stage. In human IVC, embryos reach the blastocyst stage in approximately six days, whereas it takes 10 days in marmosets. A previous study reported *in vivo* marmoset embryos reach the blastocyst stage approximately eight days after ovulation [32]. The prolonged developmental period suggested this culture condition was not optimized for marmosets. Consequently, the application of other animal embryo culture conditions would not be adequate for marmosets. Therefore feasible long-term culture conditions for marmoset embryos need further investigation. Additionally, the long incubation period of the embryos after reaching the blastocyst stage on days 9–11 probably affected the birth rate of blastocysts because all embryos were transferred on day 12 and, as described above, the embryo culture condition was not optimized, thus potentially affecting the embryo quality.

The marmoset is the only nonhuman primate that has been used to generate transgenic animals with the lentiviral system and a exogenous gene, which encoded a green fluorescent protein (GFP) and were germline-transmitted [2]. This lentiviral system is the most successful transgenic method to efficiently obtain offspring in several species [33]–[35]. However, the major drawback of this technique is the limited size of the transgene (up to 8 kb) [36]. To overcome this limitation, ICSI-Tr combined with recombinases or transposases could be a powerful technique to introduce very large DNA transgenes with relatively highly efficient integration into the host genomes [37], [38]. This ICSI-Tr technique would be applicable to the marmoset and facilitate transgenesis.

In conclusion, in the marmoset, embryos produced by ICSI using *in vitro-*matured oocytes could develop to blastocysts and neonates. Several offspring were successfully derived from embryo transfer after ICSI, which is a suitable fertilization method in marmosets.
